# Patient competence in the context of cancer: its dimensions and their relationships with coping, coping self-efficacy, fear of progression, and depression

**DOI:** 10.1007/s00520-020-05699-0

**Published:** 2020-09-02

**Authors:** Jürgen M. Giesler, Joachim Weis

**Affiliations:** 1 Section of Health Care Research and Rehabilitation Research, University of Freiburg Medical Center, Hugstetter Str. 49, D 79106 Freiburg, Germany; 2 Comprehensive Cancer Center, Department of Self-Help Research, University of Freiburg Medical Center, Freiburg, Germany

**Keywords:** Cancer, Patient competence, Coping, Self-efficacy, Fear of progression, Depression

## Abstract

**Purpose:**

Influenced in part by research on coping, patient empowerment and self-efficacy, and by patient self-help initiatives, the construct of patient competencies (PC) has been elaborated and later integrated into Germany’s National Cancer Plan. As a self-report measure of PC, the Patient Competence Questionnaire 57 (PCQ-57) has only rarely been evaluated psychometrically. Therefore, we aimed to re-examine its dimensionality and its relationships with related constructs and potential psychosocial cancer outcomes.

**Methods:**

We surveyed 424 breast, colorectal, and prostate cancer patients from clinics for oncological rehabilitation and private oncology practices who completed the PCQ-57 and self-report measures of coping, coping self-efficacy, fear of progression, and depression. Patients’ PCQ-57 responses were submitted to principal axis factor analyses. Using the original scoring keys, we computed internal consistencies (Cronbach’s *α*) and Pearson correlations between all measures.

**Results:**

Factor analyses suggested 5 of the 8 original PCQ-57 dimensions to replicate satisfactorily, their internal consistencies ranging from 0.74 to 0.88. The competency of managing distress correlated significantly, highly, and negatively with fear of progression and depression (|*r*’s| ≥ 0.43) and positively with coping self-efficacy (*r* = 0.58).

**Conclusion:**

The results support the reliability and validity of 5 of the 8 original PCQ-57 scales while suggesting refinement of the others. The replicated scales may help identify patients in need of support for dealing with the multiple tasks of adjusting to cancer. Further research should clarify the conceptual and empirical relationships between PC, health literacy, and patient activation as well as potential effects of PC on psychosocial cancer outcomes.

**Electronic supplementary material:**

The online version of this article (10.1007/s00520-020-05699-0) contains supplementary material, which is available to authorized users.

## Introduction

During the course of its development as a scientific discipline [1], central goals of psycho-oncology have by now come to include supporting cancer patients as well as their family and friends through research and practice in their efforts to cope with distressing emotions associated with the diagnosis of cancer [2–5], to decide on treatment options [6–8] and to manage long-time sequelae during survivorship [9, 10]. In order to conceptualize the patient’s personal resources to meet these challenges, a variety of more general concepts are available like self-efficacy [11, 12], empowerment [13–16], patient activation [17, 18], and health literacy [19–22]. Tied more specifically to the context of cancer, the construct of patient competence (PC) has been developed on the basis of conceptual and empirical analyses [23–27] and later been integrated into Germany’s National Cancer Plan, specifying the aim of advancing PC, thus contributing to the plan’s overall goal of enhancing patient-centered cancer care [28]. In terms of a working definition, PC has been defined as a cancer patient’s *ability* to manage emotion- and problem-focused coping tasks [29, 30] arising throughout the cancer trajectory [23]. This focus on abilities links PC with the concept of self-efficacy defined as *confidence in being able to perform* specific behaviors, while distinguishing it from coping which traditionally focuses on *factual* (past or present) behaviors aimed at managing specific task and emotion-focused challenges of one’s cancer. As a multidimensional measure of PCs, the Patient Competence Questionnaire 57 (PCQ-57) has been developed with the intention to enable research on potential effects of PC on patient-reported cancer outcomes and to determine possible effects of intervention programs aimed at promoting PCs in cancer patients [23].

Although the PCQ-57 has been used as an outcome in several intervention studies [31–33], albeit showing no effect, however, information on its construct validity and reliability has never again been provided since the initial study that had reported acceptable to good internal consistencies for its scales and small to medium correlations with global health status as an indicator of quality of life [23]. In addition, the relationship of PC with conceptually related constructs and potential psychosocial cancer outcomes has not been examined thus far. Given the working definition of PC, it should therefore be especially informative to explore relationships between PC and apparently related resource constructs like cancer patients’ use of coping behaviors or their self-efficacy beliefs with respect to coping with cancer [11, 12]. Since PC may also be expected to help cancer patients manage distressing emotions, it would also be interesting to learn how PC relates to, e.g., fear of progression or depression as frequent comorbid conditions of cancer that continue to attract the interest of researchers [3, 4, 34, 35]. Such relationships would underline that PC could fruitfully be linked to concepts of self-regulation of action and emotion [12, 30, 36].

Therefore, this study intended:

- To re-examine the factor structure of the PCQ-57 and its internal consistency reliability
- To analyze its validity with respect to coping and coping self-efficacy and to fear of progression and depression.

The study was part of a larger project that additionally asked, by employing a longitudinal design with 3 measurement points (baseline, 4 weeks and 10 months post baseline), whether PC differed between patients with different types of cancer and whether it changed across time. Results from these analyses will be reported elsewhere. The present analyses are based on the baseline data from the project.

## Methods

### Participants and procedure

In line with the research questions above, participants were recruited in seven clinics for oncological rehabilitation (*n* = 377) and three private oncology practices (*n* = 47). Rehabilitation clinics were chosen, because they aim at improving physical and psychological functioning, coping with illness, self-management, and participation [37]. Oncology practices were included to ascertain recruitment of a sufficiently large number of patients under palliative treatment. To cover the most frequent tumor entities, we chose to include patients with either breast or prostate cancer. In addition, we included colorectal cancer patients to represent a tumor entity that shows a roughly comparable incidence between the sexes. Patients treated with either curative or palliative intent were both eligible for the study. Furthermore, participants were to be at least 18 years old, sufficiently fluent in German and willing to participate in a questionnaire study. Patients with acute and severe psychiatric disorders (e.g., acute psychosis) or severe cognitive impairment impeding study participation were to be excluded. Given the research questions of the project as a whole, power calculations took the intended longitudinal design into account including comparisons between the three patient groups. Assuming a small effect size *f* = 0.1, a type 1 error of 0.01, power (1 - *β*) of 0.80 and a drop-out of 25% resulted in a suggested sample size of 512 participants that should also be sufficient for the analyses reported here.

Local research coordinators (physician, study nurse, or the like) at the collaborating centers informed eligible patients about the study and asked for a written consent. Having consented, participants completed a questionnaire booklet at each of the three specified measurement points. This booklet included the PCQ-57 as well as established measures of, e.g., coping with cancer, self-efficacy for coping with cancer, fear of progression, and depression.

## Measures

### Patient competence

As a measure of PC, the *PCQ-57* includes 57 items [23]. Thirty-five of these describe problem-focused behaviors in the context of the diagnosis, treatment, and survivorship of cancer; the remaining 22 similarly address emotion-focused behaviors (cf. Tables 2 and 3). In pilot surveys, these behaviors had been rated as indicators of PC by cancer patients and psychosocial oncology experts [23]. Problem-focused items cover (a) information-seeking behaviors on diagnosis- and treatment-related topics, (b) communication with physicians, and (c) regulating one’s well-being and social relationships. Emotion-focused items represent behaviors aimed at dealing with emotional distress in the cancer context (see supplement S1 for item wordings). All PCQ items are answered on 5-point scales ranging from 1 (*not at all tru*e) to 5 (*completely true*). The emotion-focused competence items offer the additional response option “not applicable” to accommodate the possibility that a specific item may not fit the participant’s individual situation. Also different from problem-focused items, emotion-focused items have to be responded to with respect to the past 7 days to capture short-term variations that may occur when confronting emotion-focused tasks of cancer and its treatment. Averaging individual item responses, the problem-focused competence items yield five different scale scores: *seeking information* (8 items), *self-regulation* (11), *assertively interacting with physicians* (7), *striving for autonomous decisions* (7), and *interest in social services* (2)*.* Similarly, from the emotion-focused items, three scale scores are derived that measure *managing distress* (10 items), *dealing explicitly with the threat to life posed by cancer* (6), and *low avoidance* (6). High scale scores reflect a higher self-rated competence regarding the respective behavioral domain. It should be noted, however, that in the case of the *low avoidance* scale a high score actually reflects the ability *not* to make use of avoidant behaviors. The internal consistencies (Cronbach’s *α*) of the scales range from 0.64 to 0.87 [23]. Scale correlations with criteria that might be taken as preliminary indicators of concurrent validity have already been mentioned in the introductory section.

### Coping

Coping was measured with the Trier Scales of Coping (TSK) that include 37 items describing various coping behaviors [38]. Each behavior is to be rated on a 6-point scale with respect to how often it has been performed within the past weeks. Item responses are summed to give five scale scores addressing *rumination*, *searching for affiliation*, *minimizing threat*, *searching for information and social exchange*, and *searching for meaning in religion*, respectively*.* Higher scores indicate that the respective coping behaviors have been performed more frequently during the previous weeks, Cronbach’s *α* of these scales range from 0.74 to 0.88. Scale development rests on confirmatory factor analysis in cancer patients (*N* = 322). Structural invariance of the measurement model was confirmed for patients with ankylosing spondylitis (*n* = 110). Significant medium-sized correlations (|r| ≤ 0.51) of specific scales with different measures of stress management in smaller samples (*n* ≤ 117) suggest convergent validity [38].

### Self-efficacy for coping with cancer

Self-efficacy for coping with cancer was measured with the German version of the brief form of the Cancer Behavior Inventory (CBI-B-D [39]). In its currently available version, it includes the 14 items of the American short form of this instrument (CBI-B) that had later been reduced to 12 of these items, however [11]. In order to make CBI results of the present study comparable with the now established American 12-item version, the sum scores computed here are based on the 12 items of the now reduced American version. All items describe coping behaviors to be rated on a 9-point scale from 1 (*not at all confident*) to 9 (*completely confident*) with respect to how confident one feels being able to perform the behavior in question. Higher scores indicate higher coping self-efficacy. Internal consistencies are reported to be around 0.84 [11]. Across different samples, positive correlations of the CBI-B with, e.g., measures of quality of life, optimism, and satisfaction with life, and negative ones with measures of fatigue or sickness impact speak to the validity of this measure [11].

### Fear of progression

Fear of progression was measured with the short form of the Fear of Progression Inventory (FoP-Q-SF) [40]. It asks respondents to answer 12 items addressing various fears regarding the progression or relapse of a chronic life-threatening disease like cancer. The items are to be rated on a 5-point scale from never (1) to very often (5) with respect to how often each fear is experienced by the respondent. Higher scores indicate higher fear of progression. Internal consistencies are usually reported to range above 0.80 [40, 41]. Significant differences in means between groups differing by, e.g., disease stages (*p* < 0.001, *d* = 0.61) and large correlations with concurrent measures of anxiety and depression (0.71 and 0.57, respectively) support the validity of the FoP-Q-SF [41].

### Depression

Depression was measured with the PHQ-9, the German version of the depression screening module of the Patient Health Questionnaire [42]. It includes 9 symptoms of depression that are to be rated according to how often they have been experienced during the previous 2 weeks. The response scale ranges from 0 (*not at all*) to 3 (*almost every day*). Summing across symptoms yields a scale score that may range from 0 to 27. Higher scores indicate greater depression. Internal consistencies are usually reported to lie within the range of 0.88. With respect to detecting major depression, values for sensitivity and specificity of 95% and 86%, respectively, point to the criterion validity of the instrument.

### Sociodemographic and medical characteristics

A final questionnaire section asked patients to provide information on selected sociodemographic characteristics. Illness- and treatment-related data were obtained from patients’ medical records at the collaborating centers.

### Data analysis

Descriptive statistics were computed for sociodemographic and medical variables. As the overall project asked whether PC might differ by tumor entity, we compared patients on these variables by tumor site using *χ*^*2*^-techniques or ANOVAs and *ϕ* or *η*^*2*^ as effect size measures. To evaluate the factor structure of the PCQ-57 and the internal consistency of its scales, factor analyses and item analyses based on the so-called classical test theory were computed which assumes an observed test score to be a composite of an individuals’ unobservable “true score” and a random error component. Problem-focused and emotion-focused items were factored separately as in the original study [23] since they are rated with respect to different time frames and under a different response format as described above. Given the still exploratory status of research on PC, we deliberately chose to employ exploratory principal axis factor analysis followed by orthogonal varimax rotation in both cases. While carefully considering the course of eigenvalues in both these analyses, we eventually extracted 5 and 3 factors for the problem- and the emotion-focused item set respectively, as in the original analyses [23]. The sample size suggested by the overall power calculations may be considered sufficient to achieve a person to variable ratio of at least 5:1 which is frequently recommended for factor analysis [43, 44]. Missing values were not imputed. Instead, factor analyses were performed with list-wise deletion of cases with missing values in a respective item set. Interpretation of factors was guided by the size of its item loadings (*a*_*ij*_ ≥ 0.60) and the requirement that the squared loading of an item should account for at least 50% of the communality of this item (*a*_*ij*_^2^/*h*_*i.*_^2^ ≥ 0.50) [44, 45]. To judge whether item inter-correlations were appropriate for factor analysis, the Kaiser-Meyer-Olkin (KMO) measure of sampling adequacy was computed [46] which indicates the proportion of variance in a set that might be explained by underlying factors. With a possible range from 0 to 1, high KMO values indicate a variable set to be suitable for factor analysis and values higher than 0.80 are generally regarded as “meritorious.”

In order to determine the inter-correlations of the original competence scales and their relationships with coping self-efficacy, coping, fear of progression, and depression, Pearson correlations were computed. For the latter two criteria, hierarchical regressions were also computed with coping, self-efficacy, and competencies as predictors. If competencies actually contributed to an effective self-regulation of distressing emotions, one would expect negative correlations between these two criteria and positively scored competence scales like *seeking information* or *managing distress*, although these must not be mistaken to show a causal effect. All computations were performed with IBM SPSS version 23.

## Results

### Sample

In total, *N* = 424 patients could be recruited, with the proportions of breast, colorectal, and prostate cancer patients being roughly equal (see Table 1). On average, patients were *M* = 61.5 years old (*SD* = 9.5), 85% of the sample were diagnosed with cancer for their first time and median time since diagnosis was *Mdn* = 10.4 months (not in Table 1). In many of the sample characteristics in Table 1, diagnostic groups differ significantly, with effect sizes being mostly small (data not shown); however, except for age (breast cancer *M* = 58.9 years, *η*^*2*^ = 0.07), time since diagnosis (*Mdn* = 21.1 months for breast cancer women, *η*^*2*^ = 0.14), and tumor size (T1: breast cancer 43%, colorectal cancer 10%, prostate cancer 4%, *ϕ* = 0.57).

**Table 1:** Absolute (*f*) and relative (%) frequencies of sociodemographic and medical sample characteristics

Characteristic	*f*	%
Tumor diagnosis (n = 424)		
Breast cancer	145	34
Colorectal cancer	161	38
Prostate cancer	118	28
Gender (n = 424)		
Female	225	53
Male	199	47
Marital status (n = 420)		
Single	29	7
Married/Cohabitating	290	69
Divorced/separated	59	14
Widowed	43	10
Children (n = 420)		
Yes	350	83
No	70	17
Education (n = 408)		
9 years	124	30
10 years	132	32
12 years	51	13
13 years	101	25
Employment (n = 418)		
Yes (full time or other)	237	57
No	181	43
T (n = 388)		
T_1_	73	19
T_2_	126	32
T_3_	142	37
T_4_	33	8
X	14	4
N (n = 398)		
Negative	200	50
Positive	168	42
X	30	8
Primary metastases (n = 387)		
No	302	78
Yes	69	18
X	16	4
Treatment intention (n = 389)		
Curative	228	59
Palliative	133	34
X	28	7

### Factor analyses

A KMO value of 0.85 indicated the problem-focused items were well-suited for factor analysis that resulted in 10 eigenvalues > 1 (8.24, 3.33, 2.01, 1.87, 1.55, 1.45, 1.37, 1.18, 1.14, and 1.02). The intended 5-factor varimax solution (Table 2) accounts for 41% of the variance. As their loadings indicate, these factors are readily interpretable as competencies relating to *seeking information* (F-1, Table 2), *self-regulation* (F-2), *assertively interacting with physicians* (F-3), and *interest in social services* (F-4). The fifth factor (F-5) is defined by two items that cover interest in complementary medicine. Isolated cross-loadings range from 0.25 to 0.38 (*Mdn* = 0.31). As indicated by superscripts in Table 2, the two items defining factor F-5 as well as some of the items defining factor F-1 in the present analysis had loaded on the factor *striving for autonomous decisions* in the original factor analysis of the PCQ-57 [23].

**Table 2:** Varimax-rotated 5-factor solution for problem-focused items of PC in the context of cancer (*n* = 376)

Item (number and truncated wording)	F-1	F-2	F-3	F-4	F-5	h^2^
08. Took my time to discover best therapy ^a^	**0.78**					0.66
10. Sought information on pros and cons of treatments	**0.71**					0.59
01. Sought information on diagnostic procedures	**0.70**					0.53
04. Prepared for stressful diagnostic procedures	**0.70**					0.57
02. Sought information on side effects	**0.61**					0.49
03. Sought information from brochures etc.	**0.61**					0.40
09. Made the right decision for me ^a^	**0.59**		0.32			0.48
06. Asked how treatments would work	**0.47**		0.34			0.43
05. Sought information on preventing side effects	**0.**46			0.34	0.38	0.51
11. Sought second opinion ^a^	**0.43**					0.24
51. Sought information on how to cope	0.31	0.29				0.26
07. Took measures to minimize side effects	0.29			0.36	0.43	0.45
12. Doubted physicians’ recommendations ^a^	0.25					0.14
13. Left decisions to physicians ^a^	**−0.40**					0.19
48. Try to negotiate how much support I need		**0.71**				0.54
47. Make sure that others help me out		**0.67**				0.45
53. Take care to get rest and relaxation		**0.64**				0.44
52. Take care to get enough sleep		**0.55**				0.33
49. Easy to ask others for support		**0.54**				0.33
50. Feel supported by those close to me		**0.47**				0.26
55. Times of contemplation integrated in my life		**0.43**				0.29
56. Listen to what my body might want to tell me	0.26	0.39	0.36			0.36
57. Sought information on which activities to avoid	0.30	0.35				0.25
54. Examine my body for changes		0.28	0.28			0.26
22. Ask physician when I do not understand			**0.73**			0.60
23. Successful in asking physician questions			**0.65**			0.48
21. Tell physicians when I’m not content			**0.58**			0.37
20. Get wishes for treatment accepted	0.25		**0.53**			0.39
19. Tell doctors clearly when I disagree			**0.51**			0.29
18. Find it hard to describe my complaints			**−0.30**			0.13
24. Find it hard to speak my mind			**−0.31**			0.14
16. Sought information on financial support				**0.80**		0.79
17. Took efforts to obtain financial support				**0.78**		0.64
14. Sought information on complementary medicine ^a^	0.27				**0.73**	0.62
15. Contacted specialist in complementary medicine ^a^					**0.69**	0.49

Factor loadings in descending order per factor. Only loadings |*a*_*ij*_| ≥ 0.25 are printed; loadings with |*a*_*ij*_| ≥ 0.30 *and a*_*ij*_^2^/*h.*_*j*_^2^ ≥ 0.50 in bold type

^a^Item loading on the factor *striving for autonomous decision* in the original factor analysis [23]

With respect to the emotion-focused items, a KMO value of 0.79 suggested they were acceptable for factor analysis. Factor analysis resulted in 6 eigenvalues > 1 (5.16, 2.72, 1.90, 1.35, 1.11, and 1.04) with their course favoring a 3-factor solution (Table 3) that accounts for 30% of the variance. As their loadings show, these (varimax rotated) factors may be interpreted as competencies that cover *managing emotional distress* in the context of cancer (F-1, Table 2), *dealing explicitly with the threat to life posed by cancer* (F-2) and *low avoidance* (F-3). Some items show substantial double-loadings (|*a*_*ij*_| ≥ 0.30) mostly involving factors F-1 and F-2, or low commonalities (*h*^2^ ≤ 0.20). Especially factor F-3 is defined by a smaller number of items than might have been expected based on the earlier analysis [23].

**Table 3:** Varimax-rotated 3-factor solution for emotion-focused items of PC in the context of cancer (*n* = 189)

Item (number and truncated wording)	F-1	F-2	F-3	h^2^
31. Can deal with fears related to disease	**0.74**		−0.26	0.63
27. Can deal with helplessness	**0.69**		−0.27	0.56
35. Can deal with the threat caused by illness	**0.68**			0.47
36. Can deal with physical impairment	**0.62**			0.39
38. Able to accept feelings like grief	**0.60**			0.39
32. Can deal with stress of chemotherapy	**0.59**			0.36
46. Can deal with impaired mobility ^b^	**0.56**			0.33
41. Able to distract myself when thinking of disease	**0.54**			0.29
33. Can dismiss thoughts of a possible recurrence	**0.53**	−0.41		0.46
45. Know how to handle increasing pain ^b^	**0.37**	0.26		0.20
34. Hard to accept disease ^c^	**−0.47**		0.43	0.41
25. Explicitly deal with the possibility of a recurrence		**0.77**		0.60
26. Consider what disease means to my life		**0.76**		0.61
43. Consider that I might die		**0.55**		0.31
44. Try to take good care of myself	0.32	**0.36**		0.26
42. Told myself it could be worse ^a^	0.39	**−0.43**		0.37
29. Confident that all will end well ^a^	0.35	**−0.46**		0.34
39. Engage in various activities just to forget			**0.52**	0.28
37. Do not let others see how I actually feel			**0.48**	0.24
30. Feel I have to fundamentally change my life			**0.43**	0.21
28. Difficulty in expressing what I need			0.26	0.13
40. Find consolation in thinking others are worse off than me			0.25	0.11

Factor loadings in descending order per factor. Only loadings |*a*_*ij*_| ≥ 0.25 are printed; loadings with |*a*_*ij*_| ≥ 0.30 *and a*_*ij*_^2^/*h.*_*j*_^2^ ≥ 0.50 in bold type

^a, b, c^Item loading on factors 1, 2, or 3, respectively, of the original factor analysis [23]

### Internal consistency and scale inter-correlations

Except for *dealing explicitly with threat* and *low avoidance*, the internal consistencies of the PC scales based on the original scoring keys [23] are above 0.70 with a maximum of 0.88 (Table 4). Correlations between competencies are mostly significant, positive, and low to moderate in size (*Mdn*_*r*_ = 0.24, − 0.04 ≤ *r* ≤ 0.61; see Table 4), with the exception of high correlations between *seeking information* and *striving for autonomous decisions* and between *self-regulation* and *dealing explicitly with the threat to life posed by cancer* (*r* ≥ 0.50), both suggesting some overlap between these respective constructs.

**Table 4:** Means, standard deviations, and intercorrelations of subscales of patient competencies (1–8), coping self-efficacy (9), coping (10–14), fear of progression (15), and depression (16) with internal consistencies (Cronbach’s *α*) on the main diagonal (234 ≤ *n*_M,SD_ ≤ 415; 197 ≤ *n*_corr_ ≤ 408)

	Subscale^a^	M	SD	1	2	3	4	5	6	7	8	9	10	11	12	13	14	15	16
1.	SeekInfo	3.42	0.97	(0.87)															
2.	SelReg	3.49	0.68	0.38**	(0.80)														
3.	Assert	3.92	0.69	0.40**	0.39**	(0.74)													
4.	AutDec	2.45	0.87	0.61**	0.21**	0.27**	(0.72)												
5.	SocServ	3.37	1.51	0.37**	0.19**	0.22**	0.21**	(0.88)											
6.	ManDis	3.33	0.70	0.07	0.34**	0.28**	−0.04	0.04	(0.83)										
7.	DealEx	3.49	0.73	0.36**	0.50**	0.30**	0.20**	0.21**	0.12	(0.63)									
8.	LowAvoid	3.37	0.71	−0.02	0.12*	0.25**	0.06	0.02	0.21**	−0.01	(0.52)								
9.	SelfEffic	79.40	16.48	0.16*	0.35**	0.43**	0.11*	0.13*	0.58**	0.09	0.43**	(0.88)							
10.	Rumin	27.18	7.85	0.07	0.10	−0.20**	0.05	−0.07	−0.18*	0.21**	−0.47**	−0.34**	(0.79)						
11.	SeekAffil	36.06	6.71	0.37**	0.38**	0.30**	0.20**	0.18**	0.32**	0.23**	0.12*	0.36**	0.16**	(0.76)					
12.	MinThreat	37.88	4.99	0.15**	0.39**	0.18**	−0.10*	0.04	0.38**	0.23**	−0.00	0.35**	0.20**	0.42**	(0.70)				
13.	InfoExcha	27.15	7.35	0.35**	0.36**	0.03	0.29**	0.15**	−0.14*	0.25**	−0.09	0.01	0.42**	0.42**	0.21**	(0.80)			
14.	MeanRel	8.56	4.45	0.08	0.19**	0.03	0.09	0.05	0.10	0.15*	−0.14*	0.03	0.33**	0.21**	0.22**	0.25**	(0.81)		
15.	FoP	32.41	8.12	0.14*	0.02	−0.18**	0.06	0.05	−0.43**	0.23**	−0.45**	−0.49**	0.50**	0.02	−0.05	0.23**	0.13*	(0.81)	
16.	Depress	6.80	4.51	0.03	−0.22**	−0.19**	0.10	0.05	−0.46**	**0.04**	−0.43**	−0.50**	0.27**	−0.27**	−0.17**	0.05	0.07	0.46**	(0.82)

^*^ *p* ≤ 0.05. ^**^ *p* ≤ 0.01. Significant correlations in bold type. ^a^ *SeekInfo =* seeking information (as a competence); *SelReg =* self-regulation; *Assert =* assertively interacting with physicians; *AutDec =* striving for autonomous decisions; *SocServ =* interest in social services. *ManDis =* managing distress; *DealEx =* dealing explicitly with the threat to life posed by cancer; *LowAvoid =* low avoidance; *SelfEffic =* self-efficacy for coping with cancer; *Rumin =* rumination; *SeekAffil =* seeking affiliation; *MinThreat =* minimizing threat; *InfoExcha =* searching for information and social exchange; *MeanRel =* searching for meaning in religion; *FoP =* fear of progression; *Depress =* depression

Considering the relationships of PC with other constructs focusing upon cancer patients’ resources, the competencies of *managing distress*, *low avoidance*, and *being assertive with physicians* and *self-regulation* correlate significantly and moderately to highly (0.35 ≤ *r* ≤ 0.58) with self-efficacy for coping with cancer (Table 4). Thus, higher competency in these domains co-varies to some degree with higher coping self-efficacy. Similarly, given their significant and moderately high correlations, a higher competency of *self-regulation* is associated with using coping behaviors like *seeking affiliation*, *minimizing threat*, and *searching for information and social exchange* more frequently. The same is true of competency in *seeking information* which correlates moderately and significantly with using the coping behaviors of *searching for information and social exchange* and *seeking affiliation more frequently*. Furthermore, the emotion-focused competency *of managing distress* is significantly associated with more frequent use of the coping behaviors *seeking social affiliation* and *minimizing threat*. The competency of *low avoidance* correlates moderately highly and negatively (reflecting its scoring direction) with less use of *rumination* as a coping behavior. Finally, higher *self-efficacy for coping with cancer* is significantly associated with more frequent use of *seeking affiliation* and less frequent use of *rumination*, and *minimizing threat* (|*r*| ≥ 0.34).

### Correlations with fear of progression and depression

Table 4 also shows the two emotion-focused competencies of *managing distress* and *low avoidance* to correlate significantly, negatively, and moderately with *fear of progression* and *depression* (|*r*| ≥ 0.43), that is, higher competency in *managing emotional distress* and *low avoidance* are associated with *less fear of progression* and *depression*. Similarly, higher *self-efficacy for coping with cancer* is significantly and strongly associated with *less fear of progression* and *depression* (|*r*| ≥ 0.49). In contrast, high *rumination,* as a coping behavior measured with the TSK [38], is significantly and strongly associated with *greater fear of progression* (*r* = 0.50) and significantly, but to a lower extent with *less depression* (*r* = 0.27). The hierarchical regressions (not given in detail here) show *managing distress* as an independent predictor of *less* fear of progression (*β* = −0.29, _*adj*_*R*^*2*^ = 0.45, each *p* < 0.01) and *depression* (*β* = −0.24, _*adj*_*R*^*2*^ = 0.36, each *p* < 0.01).

## Discussion

This study examined psychometric properties of the PCQ-57 by analyzing its factor structure, reliability, and validity in terms of its relationships with coping, coping self-efficacy, fear of progression, and depression in a sample of breast, colorectal, and prostate cancer patients. As shown, the competencies of *seeking information*, *self-regulation*, *assertively interacting with physicians, interest in social services*, and *managing distress* suggested by the original study [23] were rather clearly represented in the factor analyses. Although some items showed double and cross-loadings, these tended to be negligible and do not contradict the interpretation of these five factors. Thus, the factor analyses generally support the multi-dimensional conceptualization of PC that guided the construction of the PCQ-57. At the same time, these analyses offer only weak support for the competencies of *dealing explicitly with the threat to life posed by cancer* and *low avoidance* to be sufficiently clearly represented in the PCQ-57 and they fail to identify the competency of *striving for autonomous decisions*.

The item-analytic results for the PCQ 57 scales mirror those of the factor analyses: competencies that tended to replicate in factor analysis show internal scale consistencies that are either satisfactory or higher compared with competencies that did not. Interestingly, except for *low avoidance*, the internal consistencies of most scales do not differ markedly from those reported in the original study [23].

Looking at the relationships of PCs with coping behaviors and coping self-efficacy, it appears noteworthy that the problem- and emotion-focused competencies measured with the PCQ-57 correlate mostly moderately highly with measures of coping and coping self-efficacy. This tends to support the assumption that these constructs share a focus on an individual’s resources, while at the same time suggesting that competencies measured with the PCQ-57 may in part capture additional facets of dealing with the challenges of one’s cancer diagnosis, treatment and survivorship. If so, this would lend further support to the construct validity of this measure.

Turning to *concurrent* validity, it is worth noting that the emotion-focused competencies of *managing distress* and—in spite of its weak internal consistency—*low avoidance* correlates substantially with fear of progression and depression. This suggests that emotion-focused competencies may in fact play a role in the self-management of emotional distress in cancer patients, thus linking the concept to broader theories of self-regulation [12, 30, 36]. This finding also extends our knowledge of correlates of distressing emotions in cancer for which associations with specific coping behaviors [47] and coping self-efficacy [48] have been demonstrated in recent systematic reviews although some heterogeneity between studies had to be acknowledged. It remains to be seen, however, whether PCs will provide a unique contribution to the prediction and explanation of fear of progression or depression. Also, and similar to the fields of coping and coping self-efficacy, testing directional effects of PC on emotion-related cancer outcomes will require further research using longitudinal designs [47, 48].

That most items defining the previous factor s*triving for autonomous decision* [23] now load on *seeking information* represents the least expected finding of this study. It may reflect a sample bias as the present study includes higher percentages of male, colorectal and prostate cancer patients than the questionnaire construction sample [23] and no patients from acute inpatient or follow-up care. However, the partial fusion of these previously distinct factors in this study that also is mirrored in high scale correlations and similar to the original study [23] may also indicate a reciprocal relationship between *seeking information* and *striving for autonomous decision*, that is, acquiring information may facilitate autonomous decisions, while striving for autonomy may in turn motivate information seeking.

### Limitations

While the large sample size, the multi-center recruitment, and the comprehensive validity analyses of the PCQ-57 scales in a network of relevant psycho-oncological constructs represent strengths of this study, there are also some limitations. Firstly, the majority of the participants came from rehabilitation clinics, while patients from other settings of cancer care were less well represented. Secondly, the study included only three different tumor entities, thus restricting generalization of the results. Thirdly, it is difficult to determine the extent of a possible selection bias because the return rate has not been documented. Lastly, analyses at the subgroup level of, e.g., tumor sites would have provided further information, but were not carried out here because of the comparatively smaller sample sizes that would have resulted at this level.

## Conclusions

Summing up then, this study suggests that the PCQ-57 provides a reliable and valid measure of 5 distinct dimensions of PC in the context of cancer as intended: *seeking information*, *self-regulation*, *assertively interacting with physicians, interest in social services*, and *managing distress*. Their corresponding scales may be useful for evaluating cancer patients’ personal resources for confronting emotional distress and specific coping tasks arising from cancer and its treatment. Especially *managing distress* may represent a scale covering skills that may help reduce distress. These 5 scales also may help identify which competencies a patient may need to develop. In addition, they may be used for research on factors that might influence PC and on effects that PC may have on quality of life, emotional distress, and other cancer outcomes. The extent to which these scales may also help evaluate effects of interventions aimed at promoting PC [32, 33] clearly needs to be determined by further research, however. Finally, with respect to those 3 of the 8 PCQ-57 scales for which no strong support was found in the present analyses, it may be possible to retain *striving for autonomous decisions* after careful revision, while perhaps omitting *dealing explicitly with the threat to life* and *low avoidance* from the instrument. Given the currently renewed and intensified interest in health literacy in cancer [21, 22], another important task of future research will probably be clarifying its conceptual and empirical relationships with other frequently invoked resource constructs like empowerment, patient activation, or PC, and their contributions to psychosocial cancer outcomes.

## Electronic supplementary material

ESM 1 (DOCX 18.7 kb)

## References

[1] Holland JC (1992). Psycho-oncology: overview, obstacles and opportunities. Psycho-Oncology.

[2] Simard S, Thewes B, Humphris G, Dixon M, Hayden C, Mireskandari S, Ozakinci G (2013). Fear of cancer recurrence in adult cancer survivors: a systematic review of quantitative studies. J Cancer Surviv.

[3] Smith AB, Sharpe L, Thewes B, Turner J, Gilchrist J, Fardell JE, Girgis A, Tesson S, Descallar J, Bell ML, Beith J, Butow P, Beatty L, Bennett B, Brebach R, Brock C, Butler S, Byrne D, Day S, Diggens J, Fairclough A, Faulkner T, Ftanou M, Grier M, Hill G, Jones T, Kirsten L, McConaghey S, McKinnon S, Mihalopoulos C, Mireskandari S, Musiello T, Penhale J, Pollard A, Rangganadhan A, Scealy M, Scott M, Shih S, Teoh M, Tiller K, Watt P, the ConquerFear Authorship G (2018). Medical, demographic and psychological correlates of fear of cancer recurrence (FCR) morbidity in breast, colorectal and melanoma cancer survivors with probable clinically significant FCR seeking psychological treatment through the ConquerFear study. Support Care Cancer.

[4] Vehling S, Koch U, Ladehoff N, Schön G, Wegscheider K, Heckl U, Weis J, Mehnert A (2012). Prevalence of affective and anxiety disorders in cancer: systematic literature review and meta-analysis (Prävalenz affektiver und Angststörungen bei Krebs: Systematischer Literaturreview und Metaanalyse). Psychother Psychosom Med Psychol.

[5] Mehnert A, Vehling S, Scheffold K, Ladehoff N, Schön G, Wegscheider K, Heckl U, Weis J, Koch U (2013). Prevalence of adjustment disorder, acute and posttraumatic stress disorders as well as somatoform disorders in cancer patients (Prävalenz von Anpassungsstörung, Akuter und Posttraumati-scher Belastungsstörung sowie somatoformen Störungen bei Krebspatienten). Psychother Psychosom Med Psychol.

[6] Gattellari M, Voigt KJ, Butow PN, Tattersall MHN (2002). When the treatment goal is not cure: are cancer patients equipped to make informed decisions?. J Clin Oncol.

[7] Kane HL, Halpern MT, Squiers LB, Treiman KA, McCormack LA (2014). Implementing and evaluating shared decision making in oncology practice. CA Cancer J Clin.

[8] Kashaf MS, McGill E (2015). Does shared decision making in cancer treatment improve quality of life? A systematic literature review. Med Decis Mak.

[9] Rowland JH, Kent EE, Forsythe LP, Loge JH, Hjorth L, Glaser A, Mattioli V, Fossa SD (2013). Cancer survivorship research in Europe and the United States: where have we been, where are we going, and what can we learn from each other?. Cancer.

[10] Kiserud CE, Dahl AA, Fosså SD, Goerling U, Mehnert A (2018). Cancer survivorship in adults. Psycho-oncology.

[11] Heitzmann CA, Merluzzi TV, Jean-Pierre P, Roscoe JA, Kirsh KL, Passik SD (2011). Assessing self-efficacy for coping with cancer: development and psychometric analysis of the brief version of the cancer behavior inventory (CBI-B). Psychooncology.

[12] Merluzzi TV, Philip EJ, Heitzmann Ruhf CA, Liu H, Yang M, Conley CC (2018). Self-efficacy for coping with cancer: revision of the cancer behavior inventory (version 3.0). Psychol Assess.

[13] Aujoulat I, d'Hoore W, Deccache A (2007). Patient empowerment in theory and practice: polysemy or cacophony?. Patient Educ Couns.

[14] Groen WG, Kuijpers W, Oldenburg HS, Wouters MW, Aaronson NK, van Harten WH (2015). Empowerment of cancer survivors through information technology: an integrative review. J Med Internet Res.

[15] Eskildsen NB, Joergensen CR, Thomsen TG, Ross L, Dietz SM, Groenvold M, Johnsen AT (2017). Patient empowerment: a systematic review of questionnaires measuring empowerment in cancer patients. Acta Oncol.

[16] McCorkle R, Ercolano E, Lazenby M, Schulman-Green D, Schilling LS, Lorig K, Wagner EH (2011). Self-management: enabling and empowering patients living with cancer as a chronic illness. CA Cancer J Clin.

[17] Hibbard JH, Stockard J, Mahoney ER, Tusler M (2004). Development of the patient activation measure (PAM): conceptualizing and measuring activation in patients and consumers. Health Serv Res.

[18] Hibbard JH, Mahoney E, Sonet E (2017). Does patient activation level affect the cancer patient journey?. Patient Educ Couns.

[19] Sorensen K, Van den Broucke S, Fullam J, Doyle G, Pelikan J, Slonska Z, Brand H, Consortium Health Literacy Project European (2012). Health literacy and public health: a systematic review and integration of definitions and models. BMC Public Health.

[20] Osborne RH, Batterham RW, Elsworth GR, Hawkins M, Buchbinder R (2013). The grounded psychometric development and initial validation of the health literacy questionnaire (HLQ). BMC Public Health.

[21] Altin SV, Halbach S, Ernstmann N, Stock S (2015). How can we measure cancer literacy?--a systematic review on the quality of available measurement tools. Z Evid Fortbild Qual Gesundhwes.

[22] Goodwin BC, March S, Zajdlewicz L, Osborne RH, Dunn J, Chambers SK (2018). Health literacy and the health status of men with prostate cancer. Psychooncology.

[23] Giesler JM, Weis J (2008). Developing a self-rating measure of patient competence in the context of oncology: a multi-center study. Psychooncology.

[24] Weis J, Giesler JM (2008). Subjective dimensions of patient competence: relationships with selected healthcare usage behaviors and general features of self-rated competence. Patient Educ Couns.

[25] Kösters W (2000). Self-help in action. Becoming a successful patient.

[26] Rogers A, Kennedy A, Bower P, Gardner C, Gately C, Lee V, Reeves D, Richardson G (2008). The United Kingdom expert patients programme: results and implications from a national evaluation. Med J Aust.

[27] Hoffman B, Stovall E (2006). Survivorship perspectives and advocacy. J Clin Oncol.

[28] Federal Ministry of Health (2017). National Cancer Plan. Fields of action, goals, recommendations for implementation and results.

[29] Lazarus RS (1993). Coping theory and research: past, present, and future. Psychosom Med.

[30] Folkman S, Greer S (2000). Promoting psychological well-being in the face of serious illness: when theory, research and practice inform each other. Psychooncology.

[31] Giesler JM, Keller B, Repke T, Leonhart R, Weis J, Muckelbauer R, Rieckmann N, Muller-Nordhorn J, Lucius-Hoene G, Holmberg C (2017). Effect of a website that presents patients’ experiences on self-efficacy and patient competence of colorectal cancer patients: web-based randomized controlled trial. J Med Internet Res.

[32] Klafke N, Mahler C, Uhlmann L, von Hagens C, Bentner M, Schneeweiss A, Müller A, Szecsenyi J, Joos S (2018). Effects of a supportive nurse-led program including complementary and integrative medicine (CIM) on quality of life and patient competence in gynecologic cancer patients undergoing chemotherapy – results of a randomized controlled trial. Oncol Res Treat.

[33] Weis JB, Gschwendtner K, Giesler JM, Adams L, Wirtz MA (2019). Psychoeducational group intervention for breast cancer survivors: a non-randomized multi-center pilot study. Support Care Cancer.

[34] Butow P, Shaw J, Vaccaro L, Sharpe L, Dhillon H, Smith B, PoCo GFCRIG (2019). A research agenda for fear of cancer recurrence: a Delphi study conducted in Australia. Psycho-oncology.

[35] Korsten LHA, Jansen F, de Haan BJF, Sent D, Cuijpers P, Leemans CR, Verdonck-de Leeuw IM (2019). Factors associated with depression over time in head and neck cancer patients: a systematic review. Psychooncology.

[36] Carver CS, Scheier MF (1998). On the self-regulation of behavior.

[37] Bitzer EM, Dierks ML, Heine W, Becker P, Vogel H, Beckmann U, Butsch R, Dorning H, Bruggemann S (2009). Empowerment and health literacy in medical rehabilitation - recommendations for strengthening patient education. Rehabilitation.

[38] Klauer T, Filipp SH (1993). The Trier scales of coping with illness.

[39] Giesler JM, Reuter K, Weis J (2006) Changes in self-efficacy for coping with cancer during oncological rehabilitation – a study applying the German brief form of the Ccncer behavior inventory. In: DRV-Bund (ed) 18. Rehabilitationswissenschaftliches Kolloquium, Münster. Rehabilitationswissenschaftliches Kolloquium, vol 83. Berlin pp 268-269

[40] Mehnert A, Herschbach P, Berg P, Henrich G, Koch U (2006). Fear of progression in breast cancer patients--validation of the short form of the Fear of Progression Questionnaire (FoP-Q-SF). Z Psychosom Med Psychother.

[41] Hinz A, Mehnert A, Ernst J, Herschbach P, Schulte T (2015). Fear of progression in patients 6 months after cancer rehabilitation. A validation study of the fear of progression questionnaire FoP-Q-12. Support Care Cancer.

[42] Kocalevent RD, Hinz A, Brahler E (2013). Standardization of the depression screener patient health questionnaire (PHQ-9) in the general population. Gen Hosp Psychiatry.

[43] Gorsuch RL (1983). Factor analysis.

[44] MacCallum RC, Widaman KF, Zhang S, Hong S (1999). Sample size in factor analysis. Psychol Methods.

[45] Fürntratt E (1969). Determining the number of interpretable common factor in factor analyses of psychological data. Diagnostica.

[46] Kaiser HF, Cerny BA (1977). A study of a measure of sampling adequacy for factor-analytic correlation matrices. Multivar Behav Res.

[47] Morris N, Moghaddam N, Tickle A, Biswas S (2018). The relationship between coping style and psychological distress in people with head and neck cancer: A systematic review. Psychooncology.

[48] Chirico A, Lucidi F, Merluzzi T, Alivernini F, Laurentiis MD, Botti G, Giordano A (2017) A meta-analytic review of the relationship of cancer coping self-efficacy with distress and quality of life. Oncotarget 8(22):36800–36811 10.18632/oncotarget.15758 PMC5482699 28404938

