# Supplementary material for: Patient competence in the context of cancer: its dimensions and their relationships with coping, coping self-efficacy, fear of progression, and depression
Source: Support Care Cancer. 2020 Sep 2;29(4):2133–43. doi: 10.1007/s00520-020-05699-0 (PMC7892518; doi:10.1007/s00520-020-05699-0)
Supplement: Supplementary file 1 — (DOCX 18.7 kb) [file 520_2020_5699_MOESM1_ESM.docx]

**Scales and Items of the Patient Competence Questionnaire 57**

**A Self-Rating Measure of Patient Competence in the Context of Cancer**

**Scales and items pertaining to problem-focused competencies**

**Seeking Information (8 items)**

I sought information on diagnostic procedures

I sought information on possible side effects of treatment like nausea, vomiting, or feeling tired

I sought information about treatment in brochures, books etc.

I prepared myself well for upcoming stressful diagnostic procedures.

I sought information on how I might be able to prevent side effects of chemo- or radiation therapy

I asked the physicians about how different treatments would work

I took measures to minimize side effects from chemo- or radiotherapy like nausea, vomiting or skin reactions

I made sure to get all the relevant information on the pros and cons of different treatment options

**Self-Regulation (11 items)**

I make sure that others help me out

I discuss the amount of support I need with my family or those close to me

It is easy for me to ask others for their support.

I feel supported by those close to me with respect to my disease

I sought information on how to cope with daily hassles

I take care to get enough sleep

I take care to get sufficient rest and relaxation

I examine my body for changes or signs of possible disease

There are times of contemplation integrated in my life

I try to listen to what my body might want to tell me

I sought information on which activities I should avoid

**Assertively interacting with physicians (7 items)**

I find it hard to describe my complaints to the physicians precisely (reverse scoring)

When I disagree with a treatment the doctor suggested, I clearly tell him so

I usually succeed in getting the doctor to accept my wishes for treatment

When I’m not content with the way the doctor is treating me, I tell him so.

If there’s something the doctor is saying that I don’t understand, I ask him to explain

I succeeded in asking the doctor all the questions I intended to ask

I find it hard to speak my mind when talking to physicians (reverse scoring)

**Striving for Autonomous Decisions (7 items)**

I took the time I needed to discover which treatment is best for me

I succeeded in arriving at a decision that was right for me

In making a decision I consulted another physician, i.e. I sought a “second opinion”

I had my doubts with respect to the treatments the physicians suggested

I left decisions concerning my treatment to the physicians

I sought information on “unconventional therapies” (i.e. natural healing or complementary medicine)

I involved a specialist in “unconventional” therapies in treating my disease (e.g. homeopathy, traditional Chinese medicine)

**Interest in Social Services (2 items)**

I sought information on the availability of financial support for my cancer therapy

I took efforts to obtain financial or other support for my cancer

**Scales and items pertaining to emotion-focused competencies.**

(Items to be answered with reference to the previous 7 days)

**Managing distress (10 items)**

I can deal with feelings of helplessness

I am confident that all will end well

I can cope with fears related to my illness

I can deal with the stress of chemo- or radiotherapy

I can dismiss thoughts of a possible recurrence

I can deal with the threat caused by my illness

I can deal with lasting physical impairment resulting from my illness

I can manage to accept feelings like grief, fear, or anger stemming from my illness

I am able to distract myself when being distressed by thoughts about my disease

With respect to my disease I tell myself it could be worse

**Dealing explicitly with the threat to life posed by cancer (6 items)**

I explicitly deal with the possibility of a recurrence

I consider what my disease means to my future life

I consider the possibility that I might die

I try to take good care of myself

I know how to handle an increase in pain caused by my illness

I can deal with impaired physical motility

**Low Avoidance (6 items)**

I find it difficult expressing my needs or wishes to others (reverse scoring)

I feel like I have to fundamentally change my life (reverse scoring)

I find it hard to accept my disease (reverse scoring)

I don’t let others see how I actually feel (reverse scoring)

I engage in various activities just to forget about my disease (reverse scoring)

I find consolation in thinking others are worse off than me (reverse scoring)
